# Drug development concerning metallo-β-lactamases in gram-negative bacteria

**DOI:** 10.3389/fmicb.2022.959107

**Published:** 2022-09-15

**Authors:** Xiuyun Li, Jing Zhao, Bin Zhang, Xuexia Duan, Jin Jiao, Weiwei Wu, Yuxia Zhou, Hefeng Wang

**Affiliations:** ^1^Maternal and Child Health Development Research Center, Shandong Provincial Maternal and Child Health Care Hospital, Jinan, China; ^2^Pharmaceutical Department, Shandong Provincial Taishan Hospital, Taian, China; ^3^Department of Ophthalmology, Shandong Provincial Maternal and Child Health Care Hospital, Jinan, China; ^4^Physical Examination Center, Shandong Provincial Maternal and Child Health Care Hospital, Jinan, China; ^5^Department of Clinical Laboratory, Shandong Provincial Maternal and Child Health Care Hospital, Jinan, China; ^6^Department of Pediatric Surgery, Shandong Provincial Maternal and Child Health Care Hospital, Jinan, China

**Keywords:** antibiotic resistance, metallo-β-lactamases, novel drug strategies, *Escherichia coli*, *Klebsiella pneumoniae*, *Pseudomonas aeruginosa*, *Acinetobacter baumannii*

## Abstract

β-Lactams have been a clinical focus since their emergence and indeed act as a powerful tool to combat severe bacterial infections, but their effectiveness is threatened by drug resistance in bacteria, primarily by the production of serine- and metallo-β-lactamases. Although once of less clinical relevance, metallo-β-lactamases are now increasingly threatening. The rapid dissemination of resistance mediated by metallo-β-lactamases poses an increasing challenge to public health worldwide and comprises most existing antibacterial chemotherapies. Regrettably, there have been no clinically available inhibitors of metallo-β-lactamases until now. To cope with this unique challenge, researchers are exploring multidimensional strategies to combat metallo-β-lactamases. Several studies have been conducted to develop new drug candidates or calibrate already available drugs against metallo-β-lactamases. To provide an overview of this field and inspire more researchers to explore it further, we outline some promising candidates targeting metallo-β-lactamase producers, with a focus on *Escherichia coli, Klebsiella pneumoniae, Pseudomonas aeruginosa*, and *Acinetobacter baumannii*. Promising candidates in this review are composed of new antibacterial drugs, non-antibacterial drugs, antimicrobial peptides, natural products, and zinc chelators, as well as their combinations with existing antibiotics. This review may provide ideas and insight for others to explore candidate metallo-β-lactamases as well as promote the improvement of existing data to obtain further convincing evidence.

## Introduction

β-Lactams, mainly penicillins, cephalosporins, carbapenems, and monobactams, are the most important and frequently used classes of antibiotics in medicine and play a vital role in the treatment of severe gram-negative infections. They inhibit the synthesis of bacterial cell walls by mimicking the natural substrates of transpeptidase and preventing cross-linking of adjacent peptidoglycan strands (Bush and Bradford, 2016). In recent years, however, the efficacy of antibiotics has been threatened by a global rise in drug resistance, of which resistance to β-lactams is mainly mediated by β-lactamases (Eurosurveillance Editorial, 2015). Carbapenem-resistant Enterobacteriaceae, carbapenem-resistant *Pseudomonas aeruginosa* (*P. aeruginosa*), and carbapenem-resistant *Acinetobacter baumannii* (*A. baumannii*) have been listed by the World Health Organization (WHO) as pathogens that urgently need to be studied in research aiming to develop new antibiotics because of the emergence of these drug-resistant pathogens; due to the limited number of treatments, these resistant bacteria pose a serious public health problem (Tangden and Giske, 2015; WHO, 2017).

β-lactamases fall into four categories, namely, Ambler classes A, B, C, and D. Classes A, C, and D feature active-site serine β-lactamases (SBLs), and class B contains zinc-dependent metallo-β-lactamases (MBLs) (Tooke et al., 2019). MBLs require one or two zinc ions for enzyme activity and are further divided into three subclasses (i.e., B1, B2, and B3), based primarily on their metal content and the characteristics of different active sites (Boyd et al., 2020). Among antimicrobial resistance, the expression of β-lactamases, especially MBLs, is of particular interest (Boyd et al., 2020). Most MBLs that have been identified in clinical isolates thus far belong to subclass B1, among which the New Delhi metallo-β-lactamase (NDM), Verona imipenemase (VIM), and imipenemase (IMP) families are the three most common types (Hansen, 2021). MBLs are a particular challenge for several reasons: (i) They have the ability to hydrolyze and develop resistance to virtually all existing β-lactam antibiotics; (ii) there are almost no clinically useful drug regimens for MBLs; (iii) there is a rapid pace of isolation and discovery of new varieties; (iv) their coding genes are easily transferred; and (v) they are ubiquitous in hospitals and the natural environment. Over the past decade, great progress has been made in antibiotic development. Newly approved combinations of β-lactam/β-lactamase inhibitors, such as ceftazidime-avibactam (CAZ-AVI), have emerged as new options for the treatment of drug-resistant bacterial infections related to β-lactamases (Shirley, 2018). However, these new antibiotics only have activity against SBLs, not MBLs, making the fight against MBLs a challenging public health problem (Boyd et al., 2020). Infections caused by MBL-positive isolates not only contribute significantly to increased patient morbidity and mortality, length of hospital stay, and medical costs but also face limited therapeutic options (Boyd et al., 2020). Moreover, the current COVID-19 pandemic further aggravated this scenario (Falcone et al., 2021b; Westblade et al., 2021). Several recent reports have described that COVID-19 patients have an increased risk of MBL-CRE acquisition compared with usual patients, adding to the challenge of managing COVID-19 patients (Farfour et al., 2020; Nori et al., 2020; Porretta et al., 2020). Nori et al. reported five cases of NDM-producing Enterobacterales infections in New York City COVID-19 patients, which may be the first reported cases of NDM emergence in COVID-19 patients (Nori et al., 2020). An outbreak of NDM-5-producing *Escherichia coli* (*E. coli*) in a French hospital department dedicated to COVID-19 patients were described, which may be related to the following factors: the multiple-bedroom configuration of the department, uncomplete compliance for standard and contact precautions, overwork due to the burden of the disease, lack of training of staff for the care of ICU patients, and misuse of gloves (Farfour et al., 2020). In an Italian hospital, COVID-19 patients were found to have an increased risk of NDM-CRE acquisition versus the usual patients (75.9 vs. 25.3 cases/10,000 patient days) (Porretta et al., 2020).

Considering that no MBL inhibitors are clinically available to date, there is an urgent and unmet medical need for the development of therapies targeting MBL-producing pathogens. In this study, we summarize the major advances in the development of alternative drugs concerning B1 MBLs (mainly NDM, VIM, and IMP) in Enterobacteriaceae and non-fermenting gram-negative bacteria, with a focus on *E. coli* and *Klebsiella pneumoniae* (*K. pneumoniae*) for the former, as well as a focus on *P. aeruginosa* and *A. baumannii* for the latter. The *in vitro* activity and *in vivo* efficacy of novel drugs or compounds vs. MBL producers adapted are summarized in Tables 1, 2, respectively.

**Table 1:** *In vitro* activity of novel drug strategies vs. metallo-β-lactamase producers adapted from references.

**Target drugs or compounds ^a^**	**MIC ^b^ (μg/mL)**	**IN ^c^**	**Strains and numbers ^d^**	**Genotypes of metallo-β-lactamase ^e^**
Cefiderocol (Jacobs et al., 2019)	8^#^	NA	Eb, *n =* 267/267	NDM
Cefiderocol (Ito et al., 2018)	0.125–2	NA	PA, *n =* 173/452	IMP-1/-6, NDM-1+SBL
	0.125–2	NA	PA, *n =* 4/4	IMP-1, VIM-1/-2/-6
	0.125	NA	AB, *n =* 1/1	IMP-1
Cefiderocol (Kazmierczak et al., 2019)	0.12–8	NA	Eb, *n =* 39/39	VIM, NDM
	0.008–2	NA	PA, *n =* 30/30	IMP, VIM
	1	NA	AB, *n =* 2/2	NDM
Cefiderocol (Dobias et al., 2017)	4^#^	NA	Eb, *n =* 134/134	NDM-1/-4/-5/-6/-7, VIM-1/-2-/-4/-19, IMP-1/-4/-8
	2^#^	NA	PA, *n =* 30/30	IMP-1/-2/-10/-13/-15/-19/-29, VIM-1/-2, SPM-1, GIM-1
	4^#^	NA	AB, *n =* 2	NDM-1, IMP-4
Cefiderocol (Ghebremedhin and Ahmad-Nejad, 2021)	0.016–2 0.016–2	NA NA	AB, *n =* 5/7 Eb, *n =* 6/11	GIM-1, NDM-1+NDM-6, NDM-2/-9, NDM-1+NDM-6+SBL NDM-3/-5, NDM-1+NDM-6, NDM-1+NDM-6+NDM-16, NDM-1+NDM-6+NDM-16+SBL, NDM-1+NDM-6+SBL, NDM-5+NDM-20, NDM-7+NDM-19, VIM-1+SBL, VIM-1/-2/-4
	0.19–2	NA	PA, *n =* 9/9	VIM-2
Cefiderocol (Nakamura et al., 2019)	2–4	NA	Eb, *n =* 3/5	NDM-1/-4
	1–2	NA	PA, *n =* 2/2	IMP-1, VIM-10
Cefiderocol (Matsumoto et al., 2017)	2	NA	PA, *n =* 1	IMP-1
	8	NA	KP, *n =* 1	NDM-1
ATM+CAZ-AVI (Niu et al., 2020)	≤0.125+NA/4	NK	Eb, *n =* 68	MBL, MBL+ SBL
ATM+CAZ-AVI (Feng et al., 2021)	≤0.5+NA/4	SYN	Eb, *n =* 17/19	NDM-1/-5/-7/-9/-13+SBL
ATM+CAZ-AVI (Maraki et al., 2021)	NA+NA/NA	SYN	KP, *n =* 39/40	NDM-1, NDM+SBL
ATM+CAZ-AVI (Emeraud et al., 2019)	0.032–4+NA/4	NK	Eb, *n =* 43/50	NDM-1/-4/-5/-6/-7/+SBL, VIM-1/-2/-4/-9+SBL, IMP-8+SBL, GIM-1+SBL, TMB-1+SB
	6–8+NA/4	NK	PA, *n =* 2/3	VIM-1+ SBL, IMP-1+SBL, IMP-2+SBL
ATM+CAZ-AVI (Khan et al., 2021)	≤0.25–4+NA/4	SYN	Eb, *n =* 6/7	NDM-5, VIM, NDM, NDM+SBL
	4–8+4	SYN	PA, *n =* 0/6	NDM-1, VIM-2/-11, IMP-1
ATM+AVI (Karlowsky et al., 2017)	≤0.015–8+4	NK	Eb, *n =* 267/267	MBL, MBL+ SBL
	8+4	NK	PA, *n =* 173/452	MBL
ATM+AVI (Zhang et al., 2020)	0.25–4+4	NK	Eb, *n =* /161	MBL, MBL+ SBL
ATM+AVI (Biagi et al., 2019)	≤0.03–4+4	NK	Eb, *n =* 7/8	NDM+SBL
ATM+AVI (Niu et al., 2020)	≤0.25–8+4	NK	Eb, *n =* 68	MBL, MBL+SBL
ATM+AVI (Cervino et al., 2021)	≤0.25–4+4	NK	Eb, *n =* 63/64	MBL (maily NDM) +SBL
ATM+AVI (Bhatnagar et al., 2021)	0.06–4/4	NK	Eb, *n =* 55/64	NDM, NDM+SBL
ATM+AVI (Feng et al., 2021)	≤0.5–4+4	SYN	Eb, *n =* 17/19	NDM-1/-5/-7/-9/-13+SBL
CFP+VNRX-5133 (Piccirilli et al., 2021)	0.12–2+4	NK	Eb, *n =* 20/26	NDM-1/-5/-7, VIM-1/-2/-4/-19, IMP-1/-4+SBL, NDM-1+SBL
CFP+VNRX-5133 (Vazquez-Ucha et al., 2022)	≤2+4	NK	Eb, *n =* 42/56	MBL
CFP+VNRX-5133 (Hernandez-Garcia et al., 2022)	≤8+4 ≤8+4	NK NK	Eb, *n =* 14/16 PA, *n =* 30/49	NDM, VIM, IMP VIM
CFP+VNRX-5133 (Hamrick et al., 2020)	0.12–2+4	NK	EC, *n =* 5/5	VIM-1/-2/-4, SPM-1, GIM-1
	2+4	NK	EC, *n =* 0/4	NDM-1/-5/-7, IMP-1
	4+4	NK	PA, *n =* 1/1	VIM
CFP+VNRX-5133 (Kloezen et al., 2021)	1+4^#^	NK	Eb, *n =* 11/11	VIM
	32+4^#^	NK	PA, *n =* 9/9	VIM
CFP+VNRX-5133 (Mushtaq et al., 2021)	0.25–2/4	NK	Eb, *n =* 25/40	NDM
	0.06–2+4	NK	Eb, *n =* 37/40	VIM
	2+4	NK	Eb, *n =* 0/13	IMP
	1–8+4	NK	PA, *n =* 7/20	VIM
	8+4	NK	PA, *n =* 0/4	NDM, SPM
	8+4	NK	AB, *n =* 0/10	NDM
CFP+VNRX-5133 (Wang et al., 2020)	0.12–2+4	NK	Eb, *n =* 29/87	NDM
MER+VNRX-5133 (Wang et al., 2020)	0.016–1+4	NK	Eb, *n =* 59/87	NDM
ZID (Livermore et al., 2017)	≤1	NA	Eb, *n =* the great majority/35	MBL
	8–32	NK	PA, *n =* 9/10	MBL
	32	NK	AB, *n =* 0/5	MBL
CFP+ZID (Livermore et al., 2017)	2+0.06	NK	Eb, *n =* 3/35	MBL
	4+4	NK	PA, *n =* 1/10	MBL
	32+4	NK	AB, *n =* 0/5	NDM
CFP+ZID (Vazquez-Ucha et al., 2022)	≤2+ ≤ 2	NK	Eb, *n =* 54/56	MBL
CFP+ZID (Bhagwat et al., 2021)	≤0.06–2+ ≤ 0.06–2	NK	Eb, *n =* 166/186	MBL
	0.25–8+0.25–8	NK	Pseudomonas, *n =* 93/106	MBL
CFP+ZID (Karlowsky et al., 2020)	0.5–8+0.5–8^#^	NK	Eb, *n =* 214/214	NDM/VIM/IMP, NDM/VIM/IMP+SBL
	16+16^#^	NK	PA, *n =* 94/94	NDM, VIM, IMP
CFP+ZID (Yang et al., 2020)	≤2+NA	NK	Eb, *n =* 39/47	NDM
CFP+ZID (Avery et al., 2020)	0.19–2+NA	NK	Eb, *n =* 15/15	NDM-1/-5/-6/+SBL, VIM-1+SBL, IMP-4+SBL
CFP+ZID (Monogue et al., 2019)	4–8+4–8	NK	PA, *n =* 6/10	VIM-1/-2/-28, VIM-2/-49+SBL, NDM+SBL, VIM+SBL
CFP+ZID (Kidd et al., 2020)	4–8+4–8	NK	PA, *n =* 5/9	VIM-1-/-2, VIM-2/-49+SBL, NDM+SBL, VIM+SBL
CFP+ZID (Moya et al., 2017)	4–16+4	SYN	PA, *n =* 2/2	VIM-1/-2
CFP+ZID (Moya et al., 2019)	≤0.25–0.5+4	SYN	KP, *n =* 4/6	VIM-1, NDM-1+SBL, NDM+SBL,
ATM+ZID (Moya et al., 2017)	≤0.5–2+4	SYN	PA, *n =* 2/2	VIM-1/-2
ATM+ZID (Moya et al., 2019)	≤0.25–2+4	SYN	KP, *n =* 4/6	VIM-1, NDM-1+SBL, NDM+SBL
OP0595 (Livermore et al., 2015)	4	NA	Eb, *n =* 31/40	MBL
OP0595 (Mushtaq et al., 2019)	1–4	NA	KP, *n =* 27/104	NDM
ATM+OP0595 (Livermore et al., 2015)	0.03–1+1	NK	KP, *n =* 9/9	NDM, VIM, IMP
BIA+OP0595 (Livermore et al., 2015)	≤0.02–2+4	NK	KP, *n =* 6/9	
CFP+OP0595 (Livermore et al., 2015)	≤0.02–2+4	NK	KP, *n =* 6/9	
PIP+OP0595 (Livermore et al., 2015)	≤0.02–2+4	NK	KP, *n =* 8/9	
CAZ-AVI+OP0595 (Livermore et al., 2015)	2/4	NK	KP, *n =* 1/9	
MER+OP0595 (Mushtaq et al., 2019)	1+4	NK	KP, *n =* 60/104	NDM
CFP+OP0595 (Mushtaq et al., 2019)	2+4	NK	KP, *n =* 73/104	
ATM+OP0595 (Mushtaq et al., 2019)	1+4	NK	KP, *n =* 104/104	
CFP+WCK5153 (Moya et al., 2017)	1–4+4	SYN	PA, *n =* 2/2	VIM-1/-2
ATM+WCK5153 (Moya et al., 2017)	≤0.5+4	SYN	PA, *n =* 2/2	
PIP+WCK5153 (Moya et al., 2017)	4+4	SYN	PA, *n =* 1/2	
IMI+WCK5153 (Moya et al., 2017)	2+4	NK	PA, *n =* 0/2	
MER+WCK5153 (Moya et al., 2017)	≤0.5–2+4	SYN	PA, *n =* =2/2	
DOR+WCK5153 (Moya et al., 2017)	≤0.5+4	SYN	PA, *n =* 1/2	
CFP+WCK5153 (Moya et al., 2019)	≤0.25–0.5+4	SYN	KP, *n =* 4/6	VIM-1, NDM-1+SBL, NDM+SBL
ATM+WCK5153 (Moya et al., 2019)	≤0.25–4+4	SYN	KP, *n =* 5/6	
MER+ANT2681 (Zalacain et al., 2021)	≤0.06–1+8	NK	Eb, *n =* 1,003/1,687	MBL, MBL+SBL
MER+ANT2681 (Davies et al., 2020)	0.125–1+8	NK	Eb, *n =* 8/11	NDM-1, NDM-1/-4/-5/-7+SBL, VIM-1/-2, VIM-1/-2/-4/-19+SBL
MER+ANT2681 (Das et al., 2020)	0.06–1+8	NK	Eb, *n =* 4/6	NDM-1, NDM-1/-7+SBL
MER+QPX7728(Lomovskaya et al., 2022)	0.125+4^#^	NK	Eb, *n =* 36/36	MBL
MER+QPX7728(Nelson et al., 2020a)	1+8	NK	Eb, *n =* 220/224	MBL
MER+QPX7728(Lomovskaya et al., 2021)	≤8+8	NK	PA, *n =* 19/61	MBL
MER+QPX7728(Nelson et al., 2020b)	≤0.06–2+8	NK	Acinetobacter, *n =* 3/5	NDM
Eravacycline (Johnston et al., 2020)	2	NA	EC, *n =* 64/64	NDM, VIM, IMP
Eravacycline (Zhang et al., 2016)	2^#^	NA	Eb, *N =* 9/9	NDM-1+SBL, VIM-1+SBL
Eravacycline (Maraki et al., 2022)	1–4^#^	NA	KP, *n =* 46/46	NDM, VIM
Plazomicin (Maraki et al., 2022)	0.75–1.5	NA	KP, *n =* 46/46	
Plazomicin (Johnston et al., 2021)	2	NA	EC, *n =* 32/64	NDM, VIM, IMP
Plazomicin (Serio et al., 2019)	2	NA	Eb, 373/488	NDM, VIM, IMP
LYS228(Blais et al., 2018)	4	NA	Eb, 30/33	MBL
KBP-7072 (Huband et al., 2020b)	0.12–1	NA	AB, *n =* 5/5	DNM-1+SBL, IMP-1+SBL
Captopril±MER (Zhao et al., 2021)	160–320+ ≤ 1	NK	KP, *n =* 5/5	NDM-1, IMP-1/-26
A derivative of ebselen+MER (Jin et al., 2020)	4–32+4–32	SYN	Eb, *n =* 3/3	NDM-1, VIM-1, IMP-4
Thanatin (Ma et al., 2019)	1–8	NA	Eb, *n =* 7/7	NDM-1
Thanatin (Soren et al., 2015)	1–8	NA	Eb, *n =* 7/7	NDM-1
Thanatin+IMI (Ma et al., 2019)	NA+NA	SYN/ADD	Eb, *n =* 7/7	NDM-1
Thanatin+MER (Ma et al., 2019)	NA+NA	ADD	Eb, *n =* 7/7	
Novicidin+RIF (Soren et al., 2015)	NA+NA	SYN	Eb, *n =* 7/7	NDM-1
AMA +MER (King et al., 2014)	8+2	NK	Eb, Acinetobacter and Pseudomonas, *n =* 88–90% or more strains	NDM, VIM
AMA+MER (Rotondo et al., 2020)	4–64+2	NK	Eb, *n =* 33/35	NDM-1/-4/-5/-6/-7, VIM-1/-2/-7, CAM-1, DIM-1, IND-1, GIM-1,
AMA+DOR (Rotondo et al., 2020)	8–64+1	NK	Eb, n33/35	IMP-1/-7/-27, SPM-1, CphA2, L1, AIM-1
AMA+AMP (Rotondo et al., 2020)	≤0.5–64+8	NK	Eb, *n =* 13/35	NDM-1/-4/-5/-6/-7, VIM-1/-2/-7, CAM-1, DIM-1, IND-1, GIM-1, IMP-1/-7/-27, SPM-1, CphA2, L1, AIM-1
AMA+ERT (Rotondo et al., 2020)	8–64+0.5	NK	Eb, *n =* 20/27	NDM-1/-4/-5/-6/-7, VIM-1/-2/-7, CAM-1, DIM-1, IND-1, GIM-1,
AMA+IMI (Rotondo et al., 2020)	≤0.5–64+2	NK	Eb, *n =* 24/27	IMP-1/-7/-27, SPM-1, CphA2, L1, AIM-1
AMA+CTX (Rotondo et al., 2020)	≤0.5–64+1	NK	Eb, *n =* 13/27	
Emerione A+MER (He et al., 2022)	8–16+2	NK	Eb, *n =* 2/2	NDM-1
Emerione A+IMI (He et al., 2022)	16–32+1	NK	Eb, *n =* 2/2	
Emerione A+CTX (He et al., 2022)	64–128+1	NK	Eb, *n =* 2/2	
Emerione A+AMP (He et al., 2022)	64+8	NK	Eb, *n =* 1/2	
Rosmarinic acid+MER (Yu et al., 2018)	18+8	NK	Eb, *n =* 1/1	VIM-2
Salvianolic acid A+MER (Yu et al., 2018)	25+4	NK	Eb, *n =* 1/1	
TPEN+MER (Azumah et al., 2016)	4–16+0.5–1	NK	Eb, *n =* 9/12	NDM-1/-4, VIM-1, IMP-8
DPA+MER (Azumah et al., 2016)	8–32+0.5–1	NK	Eb, *n =* 7/12	
NOTA+MER (Somboro et al., 2015)	4+0.06–1	NK	Eb, *n =* 14/14	NDM-1/-4, VIM-1, IMP-1/-8
NOTA+IMI (Somboro et al., 2015)	4+0.125–1	NK	Eb, *n =* 7/14	
TACN+MER (Somboro et al., 2019)	4–8+0.06–8	SYN	Eb, *n =* 15/15	NDM-1/-4, VIM-1/-2, IMP-1/-8
Ca-EDTA+IMI (Yoshizumi et al., 2013)	32+1	NK	Eb, *n =* 2/4	NDM-1
Ca-EDTA+MER (Yoshizumi et al., 2013)	32+1	NK	Eb, *n =* 1/4	
Ca-EDTA+IMI (Aoki et al., 2010)	32+1–2	NK	PA, *n =* 3/16	VIM-2, IMP-1/-2/-7/-10
Ca-EDTA+AMK (Aoki et al., 2010)	32+16	NK	PA, *n =* 1/16	
NSPCs+NER (Farley et al., 2021)	2+ ≤ 0.25–0.5	NK	Eb, *n =* 4/4	NDM-1
ML302+MER (Brem et al., 2014)	10+ ≤ 0.25–0.5	NK	Eb, *n =* 3/3	IMP-1/-4
	10+1	NK	Eb, *n =* 0/2	NDM-1, VIM-4
KHP-3757 (Huband et al., 2020a)	0.06–0.5	NA	PA, *n =* 7/7	NDM-1, VIM-1/−2/−4/−20, IMP-7

a ATM, aztreonam; CAZ, ceftazidime; AVI, avibactam; CFP, cefepime; MER, meropenem; ZID, zidebactam; BIA, biapenem; PIP, piperacillin; IMI, imipenem; DOR, doripenem; RIF, rifampin; AMA, Aspergillomarasmine A; ERT, ertapenem; CTX, cefotaxime; AMP, ampicillin; TPEN, N, N′, N′-Tetrakis (2-pyridylmethyl) ethylenediamine; DPA, di-(2-picolyl) amine; NOTA, 1, 4, 7-triazacyclononane-1, 4, 7-triacetic acid; AMK, amikacin; NSPCs, N-sulfamoylpyrrole-2-carboxylates.

b MIC, minimal inhibitory concentration;

# represents MIC that inhibits 90% of the isolates (i.e. MIC90).

c IN, interaction interpretation; NA, not applicable; NK, not known; SYN, synergistic; ADD, additive. The fractional inhibitory concentration index (FIC) is calculated by comparing the value of the MIC of each agent alone with the combination-derived MIC. FICs <0.5 are considered to be synergistic. FICs in the 0.5 to 1.0 range are considered to be additive (Doern, 2014).

d Eb, Enterobacteriaceae; PA, P. aeruginosa; AB, A. baumannii; KP, K. pneumoniae; EC, E. coli; n = A/B represents that the effective result was only found in A strains out of B strains.

e SBL, the unified name for all genotypes of serine-β-lactamase; all other letter combinations represent various genotypes of metallo-β-lactamase.

**Table 2:** *In vivo* efficacy of novel drug strategies vs. metallo-β-lactamase producers adapted from references.

**Target regimens ^a^**	**Strains (MBL-producing) and numbers ^b^**	**Disease types, study objects, and numbers ^c^**	**Positive efficacy indicators**
Cefiderocol (2 g^*^q8 h, 1-h or 3-h infusion) continually at 1 or 2 h-94 h post infection (Matsumoto et al., 2017)	PA (IMP-1) and KP (NDM-1), *n =* 2	Respiratory tract infection, male Sprague-Dawley rats (~200 g), *n =* 49	94-h log_10_ CFU/lung
CAZ-AVI (32 mg/kg-8 mg/kg q8 h, s.c.) + ATM (32 mg /kg q8 h, s.c.) continually at 2 h-22 h post infection (Marshall et al., 2017)	KP (NDM-1+SBL), *n =* 1	Neutropenic thigh infection, female Hsd: ICR (CD-1) mice, *n =* 90	22-h log_10_ CFU/thigh
CAZ-AVI (2.5 g q8 h) + ATM (2 g q8 h) continually depending on clinical situation (Falcone et al., 2021a)	Eb (NDM, VIM), *n =* 102	Bloodstream infection, humans, *n =* 102	30-day all-cause mortality, clinical failure rate at day 14
CAZ-AVI (2 g-0.5 g t.i.d.) + ATM (2 g t.i.d.) continually for 7 days after switching therapy (Emeraud et al., 2019)	EC (NDM-5+SBL), *n =* 1	Acute pyelonephritis, man (70-year-old), *n =* 1	48-h body temperature, 48-h inflammatory syndrome, 48-h renal function
CAZ-AVI (2 g-0.5 g t.i.d.) + ATM (2 g t.i.d.) continually for 10 days after switching therapy (Davido et al., 2017)	KP (NDM-1+SBL), *n =* 1	Catheter-related infection with suppurated thrombophlebitis complicated with persistent bacteremia, man (69-year-old), *n =* 1	3-h blood cultures
CAZ-AVI (2.5 g q12 h) + ATM (2 g b.i.d.) continually for 6 weeks after switching therapy (Davido et al., 2017)	PA (NDM+SBL), *n =* 1	Pneumonia featuring large abscesses, man (55-year-old), *n =* 1	Clinical recovery
CAZ-AVI (50 mg/kg, q8 h, 3-h infusion) + ATM (50 mg/kg q8 h) continually for 2 weeks after switching therapy (Yasmin et al., 2020)	EH (NDM-1+SBL), *n =* 1	Infection caused by stem cell, boy (4-year-old with B cell precursor acute lymphoblastic leukemia), *n =* 1	Clinical recovery and microbiologic removal
CFP (2 g^*^ q8 h, 1-h infusion, s.c.) + ZID (1 g^*^ q8 h, 1-h infusion, s.c.) continually at 2–22 h post infection (Monogue et al., 2019)	PA (VIM, VIM+SBL, NDM+SBL), *n =* 6/10	Neutropenic thigh infection, female ICR mice (weighing 20 to 22 g), *n =* 150	22-h log_10_ CFU/thigh
CFP (2 g^*^ q8 h, 1-h infusion, s.c.) + ZID (1 g^*^ q8 h, 1-h infusion, s.c.) continually at 2–24 h post infection (Kidd et al., 2020)	PA (VIM, VIM+SBL, NDM+SBL), *n =* 6/9	Neutropenic lung pneumonia, specific-pathogen-free female CD-1 mice (mean weight 22.7 g; range 19.1 to 26.9 g), *n =* 270	24-h log_10_ CFU/lung
CFP (100 mg/kg q2 h, s.c.) + zidebactam (75 mg/kg q2 h, s.c.) continually at 2–24 h post infection (Moya et al., 2019)	KP (NDM+SBL), *n =* 3	Neutropenic thigh infection, male/female Swiss Albino mice (25–27 g), *n =* 108	24-h log_10_ CFU/thigh
ATM (75 mg/kg q2 h, s.c.) + ZID (75 mg/kg, q2 h, s.c.) continually at 2–24 h post infection (Moya et al., 2019)	KP (NDM+SBL), *n =* 3	Neutropenic thigh infection, male/female Swiss Albino mice (25–27 g), *n =* 108	24-h log_10_ CFU/thigh
CFP (100 mg/kg q2 h, s.c.) + WCK5153 (75 mg/kg q2 h, s.c.) continually at 2–24 h post infection (Moya et al., 2019)	KP (NDM+SBL), *n =* 3	Neutropenic thigh infection, male/female Swiss Albino mice (25–27 g), *n =* 108	24-h log_10_ CFU/thigh
ATM (75 mg/kg q2 h, s.c.) + WCK5153 (75 mg/kg q2 h, s.c.) continually at 2–24 h post infection (Moya et al., 2019)	KP (NDM+SBL), *n =* 3	Neutropenic thigh infection, male/female Swiss Albino mice (25–27 g), *n =* 108	24-h log_10_ CFU/thigh
MER (50 mg/kg q4 h s.c.) +ANT2681 (89 mg/kg q4 h, i.v.) (Das et al., 2020)	EC (NDM-1), *n =* 1	Neutropenic thigh infection, male CD1 mice (~25–30 g), *n =* 9	24-h log_10_ CFU/thigh
LYS228 (90 mg/kg q4 h, s.c.) (Weiss et al., 2019)	KP (NDM-1), *n =* 1	Neutropenic thigh infection, female CD-1 mice (18–20 g), *n =* 30	24-h log_10_ CFU/thigh
Captopril (25 mg/kg, injected to haemocoel) + MER (10 mg/kg, injected to haemocoel) at 2 h post infection (Zhao et al., 2021)	KP (NDM-1, IMP-4), *n =* 1/2	Haemocoel infection, *Galleria mellonella* larvae (300–350 mg), *n =* 154	94-h survival rate and 24-h CFU/larva
Thanatin (1, 3, or 6 mg/kg, i.p.) at 1 and 6 h post infection (Ma et al., 2019)	EC (NDM-1), *n =* 1	Sepsis, male BALB/c mice (weighing 18–22 g), *n =* 136	167-h survival rate, 23-h log_10_ CFU/tissues (blood, lung, liver, and spleen), 23-h pathological damages
Thanatin (0.1 mg/kg, i.p.) + MER (10 mg/kg, i.p.) at 1 h post infection (Ma et al., 2019)	EC (NDM-1), *n =* 1	Sepsis, male BALB/c mice (weighing 18–22 g), *n =* 112	167-h survival rate, 23-h CFU/spleen, 23-h CFU/liver
Ca-EDTA (200 mg/kg q.d., s.c.) or Ca-EDTA (300 mg/kg q.d., intranasal administration) + IMI (50 mg/kg q.d., s.c.) continually at 2–28 h post infection (Aoki et al., 2010)	PA (IMP-1), *n =* 1	Pneumonia, female BALB/c mice, *n =* 68	118-h survival rate and 2-h log_10_ CFU/lung
Ca-EDTA (200 mg/kg, s.c.) +MER/CS (25 mg/kg, s.c.) at 2 h post infection (Yoshizumi et al., 2013)	EC (NDM-1), *n =* 1	Acute lethal septicemia, mice, *n =* 20	2-h CFU/liver
Ca-EDTA (100 mg/kg, s.c.) + IMI/CS (10 mg/kg, s.c.) at 2 h post infection (Yoshizumi et al., 2013)	EC (NDM-1), *n =* 1	Neutropenic sepsis, mice	2-h CFU/liver and 2-h CFU/blood

a s.c., subcutaneous injection; i.v., intravenous injection; i.p., intraperitoneal injection; q.d.: one a day; b.i.d.: twice a day; t.i.d.: three a day; CAZ-AVI, ceftazidime-avibactam; ATM, aztreonam; CFP, cefepime; ZID, zidebactam; MER, meropenem; IMI, imipenem; CS, cilastatin sodium;

* represents that real drug doses used for animals are equivalent to these marked clinical doses.

b PA, P. aeruginosa; KP, K. pneumoniae; Eb, Enterobacteriaceae; EC, E. coli; EH, Enterobacter hormaechei; n = A/B represents that the effective result was only found in A strains out of B strains; SBL: the unified name for all genotypes of serine-β-lactamase; NDM, VIM, and IMP represent various genotypes of metallo-β-lactamase.

c Time points refer to the times after the initial therapy or switching therapy, with an exception for three indexes (30-day all-cause mortality, clinical failure rate at day 14, and length of stay), whose time points refer to the time after bloodstream infection onset.

## Novel antibiotics and β-lactamase inhibitors in preclinical stages

There are many ongoing efforts to overcome the resistance mediated by MBLs, one of the most important of which is developing new antibiotics or β-lactamase inhibitors. In this context, attention is primarily given to introducing new antibiotics and combinations of β-lactams and new β-lactamase inhibitors, which exhibit active antibacterial effects against MBL producers.

### Cefiderocol

Cefiderocol, a new siderophore cephalosporin that forms a complex with extracellular free iron, is transported through the extracellular membrane and exerts its bactericidal activity by inhibiting cell wall synthesis (Ito et al., 2018). Structurally, cefiderocol is similar to ceftazidime and cefepime, and the biggest highlight lies in the pyrrolidinium group on the C3 side chain and the carboxypropyl-oxyimino group on the C7 side chain, which confer its enhanced antibacterial activity and high stability to numerous β-lactamases of Ambler classes A to D, especially MBLs (Sato and Yamawaki, 2019).

A series of studies indicate that cefiderocol shows strong antibacterial activity against MBL-carrying Enterobacteriaceae, *P. aeruginosa*, and *A. baumannii in vitro*. Cefiderocol (0.016–8 μg/ml) has been proven to be effective against MBL-Enterobacteriaceae (Dobias et al., 2017; Matsumoto et al., 2017; Ito et al., 2018; Jacobs et al., 2019; Kazmierczak et al., 2019; Ghebremedhin and Ahmad-Nejad, 2021). Compared with MBL-carrying Enterobacteriaceae, cefiderocol has even more powerful antibacterial activity against MBL-carrying *P. aeruginosa* and MBL-carrying *A. baumannii*, with minimal inhibitory concentrations (MICs) in the range of 0.008–8 μg/ml (Dobias et al., 2017; Matsumoto et al., 2017; Ito et al., 2018; Jacobs et al., 2019; Kazmierczak et al., 2019; Nakamura et al., 2019; Ghebremedhin and Ahmad-Nejad, 2021). Moreover, Ghebremedhin et al. found that the antibacterial activity of cefiderocol against the same species of bacteria carrying different MBLs seems to be significantly different (Ghebremedhin and Ahmad-Nejad, 2021). NDM-1/-6-positive isolates (*K. pneumoniae* and *A. baumannii*) can be inhibited by cefiderocol at a concentration of ≤2 μg/ml, but this is not the case for NDM-5/-20, NDM-7/NDM-19, or NDM-9 (*K. pneumoniae* and *A. baumannii*), which exhibit MIC levels >16 μg/ml for cefiderocol (Ghebremedhin and Ahmad-Nejad, 2021). However, given the small number of strains involved in the study by Ghebremedhin et al., their results need to be interpreted with caution and studied further.

Animal models have also been used to evaluate the antibacterial activity of cefiderocol *in vivo* (Zhanel et al., 2019). In mouse models of neutropenic thigh and lung infection, cefiderocol (24–800 mg/kg, q24 h) was found to be effective against NDM-1-positive *K. pneumoniae* and IMP-1-positive *P. aeruginosa* at 24 h of treatment (Nakamura et al., 2019). In an immunocompetent rat respiratory infection model infected with NDM-1-positive *K. pneumoniae* and IMP-1-positive *P. aeruginosa*, cefiderocol (2 g q8 h, 1- or 3-h infusion) was reduced by >3 log_10_ CFU/lung below that in no drug therapy and ceftazidime (1 g q8 h, 0.5-h infusion) monotherapy (Matsumoto et al., 2017). These results indicate that cefiderocol has potent *in vivo* efficacy correlated with the *in vitro* activity against Enterobacteriaceae, *P. aeruginosa*, and *A. baumannii* producing MBLs.

Currently, cefiderocol has been approved by the Food and Drug Administration (FDA) and European Medicines Agency against different species of Enterobacteriaceae and non-fermenting gram-negative bacteria (Giacobbe et al., 2020). In summary, cefiderocol represents an interesting new option for multidrug resistance (MDR) gram-negative bacteria, and its structural stability makes it the most attractive new antibiotic currently available for the treatment of MBL producers.

### Aztreonam+CAZ-AVI and aztreonam+AVI

Aztreonam was approved by the FDA in 1986 and is the only clinically available monocyclic lactam antibiotic against MBLs due to its structural stability in the presence of MBLs (Ramsey and MacGowan, 2016). However, the majority of MBL-carrying isolates coproduce one or more SBLs, which limits its usefulness as a monotherapy (Cervino et al., 2021). CAZ-AVI is a drug in phase 3 clinical trials and has been recommended by the Infectious Diseases Society of America in combination with aztreonam for the treatment of severe infections caused by MBL-carrying Enterobacteriaceae (Tamma et al., 2021).

As shown in Table 1, *in vitro*, aztreonam (≤0.03–4 μg/ml)+CAZ-AVI (4 μg/ml of the AVI component) exerts potent synergistic effects against most tested MBL-producing Enterobacteriaceae and few MBL-producing *P. aeruginosa*, which is superior to aztreonam alone or in combination with other drug combinations, such as aztreonam +amoxillin-clavulanate and aztreonam+ceftolozane-tazobactam (Emeraud et al., 2019; Niu et al., 2020; Khan et al., 2021; Maraki et al., 2021). With further research, the synergistic effect of aztreonam+CAZ-AVI on MBL producers was proven to be mainly caused by the combination of aztreonam and AVI (Feng et al., 2021). AVI is a novel β-lactamase inhibitor that effectively inhibits class A and C carbapenems (Das et al., 2019). The theoretical basis for the inactivation of class A, C, or D β-lactamases by AVI to restore aztreonam susceptibility is supported by its high efficacy in patients with severe infections and without conventional treatment options (Li et al., 2015). Therefore, aztreonam+AVI is considered an attractive combination for the treatment of MBL-positive Enterobacteriaceae. A series of *in vitro* studies on the combined effects of aztreonam+AVI against MBLs have been carried out. Aztreonam+AVI (≤0.015–4+4 μg/ml) has strong antibacterial activity on Enterobacteriaceae producing MBLs alone or both MBLs and SBLs (Karlowsky et al., 2017; Biagi et al., 2019; Niu et al., 2020; Zhang et al., 2020; Bhatnagar et al., 2021; Cervino et al., 2021; Feng et al., 2021). Karlowsky et al. found that aztreonam+AVI (4 μg/ml+4 μg/ml) also has a good inhibitory effect on a small portion of MBL-*P. aeruginosa* isolates (63/452) (Karlowsky et al., 2017). To date, no *in vitro* data have been found on the inhibition of MBL-carrying *A. baumannii*.

Although a phase 3 clinical trial of aztreonam+AVI is underway (ClinicalTrials.gov:NCT 03329092), *in vivo* studies of aztreonam+AVI are mainly performed by two FDA-approved drugs, i.e., aztreonam and CAZ-AVI. *In vivo* efficacy studies in human or animal models validate the solid *in vitro* data documenting the potential of aztreonam+CAZ-AVI (Table 2). In the neutropenic thigh mice infection model caused by NDM-1+SBLs positive *K. pneumoniae*, CAZ-AVI (32 mg/kg−8 mg/kg q8 h) plus aztreonam (32 mg/kg q8 h) reduced 24-h log_10_ CFU/thigh by ~2–4 log_10_ CFU below that in no drug therapy, CAZ-AVI (32 mg/kg−8 mg/kg q8 h), and CAZ+aztreonam (64 mg/kg q8 h) monotherapy (Marshall et al., 2017). In a relatively large clinical study for patients with bloodstream infections due to NDM- or VIM-producing Enterobacteriaceae, 30-day all-cause mortality was 19.2% for CAZ-AVI (2.5 g q8 h) plus aztreonam (2 g q8 h) vs. 44% for regimens of other active antibiotics, including colistin (4.5 M UI q12 h), tigecycline (100 mg b.i.d.), fosfomycin (4–6 mg q6 h), gentamicin (3–5 mg/kg q.d.), and meropenem (2 g q8 h); clinical failure rate at day 14 was 25% for CAZ-AVI+aztreonam vs. 52% for regimens of other active antibiotics (Falcone et al., 2021a). For a 70-year-old man with acute pyelonephritis and infection caused by NDM-5+SBL-positive *E. coli*, body temperature, resolution of the inflammatory syndrome, and improvement of renal function were found 48 h after antimicrobial treatment was switched from a regimen comprising colistin, fosfomycin, and gentamicin for 7–10 days to CAZ-AVI (2 g−0.5 g t.i.d.) plus aztreonam (2 g t.i.d.) (Emeraud et al., 2019). Several clinical case reports also show the potent *in vivo* efficacy of CAZ-AVI plus aztreonam against MBL-producing Enterobacteriaceae and *P. aeruginosa*. For a 69-year-old man with catheter-related infection due to NDM-1+SBL-positive *K. pneumoniae*, blood cultures became negative at 3 h after antimicrobial treatment was switched from a regimen comprising colistin, imipenem, fosfomycin, levofloxacin, and amikacin for 26 days to CAZ-AVI (2 g−0.5 g t.i.d.) plus aztreonam (2 g t.i.d.) (Davido et al., 2017). For a 55-year-old man with pneumonia featuring large abscesses caused by NDM+SBL-positive *P. aeruginosa*, clinical recovery was gained quickly after antimicrobial treatment was switched from a regimen comprising amikacin and colistin to CAZ-AVI (2.5 g q12 h) plus aztreonam (2 g b.i.d.) (Yasmin et al., 2020). For a 4-year-old leukemia boy infected by NDM-1+KPC-4 positive *Enterobacter hormaechei* due to stem cell, clinical recovery and microbiologic removal were gained at 2 weeks after antimicrobial treatment being switched from a regimen comprising piperacillin-tazobactam+vancomycin or meropenem+vancomycin to CAZ-AVI (50 mg/kg, q8 h, 3-h infusion) plus aztreonam (50 mg/kg q8 h) (Yasmin et al., 2020).

Overall, available data support the combination of aztreonam and AVI (sometimes CAZ-AVI) against MBL-producing Enterobacteriaceae and *P. aeruginosa in vitro* as well as *in vivo*, although data on MBL-producing *A. baumannii* are scarce.

### Cefepime+VNRX-5133

VNRX-5133 (taniborbactam) is a new type of β-lactamase inhibitor in clinical development that inhibits important MBLs, including NDM-1 IMP-1 and VIM-1/2 (Krajnc et al., 2019; Lang et al., 2020). It should be noted that VNRX-5133 inhibited IMP less than NDM and VIM (Krajnc et al., 2019). Several *in vitro* studies have found that VNRX-5133 at 4 μg/ml can restore the antibacterial activity of cefepime against most Enterobacteriaceae producing NDM and VIM (Hamrick et al., 2020; Kloezen et al., 2021; Mushtaq et al., 2021; Piccirilli et al., 2021; Hernandez-Garcia et al., 2022; Vazquez-Ucha et al., 2022). However, cefepime+VNRX-5133 (0.12–8 μg/ml+4 μg/ml) has very weak or no antibacterial activity against Enterobacteriaceae-producing IMP (Hamrick et al., 2020; Wang et al., 2020; Kloezen et al., 2021; Mushtaq et al., 2021; Piccirilli et al., 2021; Hernandez-Garcia et al., 2022; Vazquez-Ucha et al., 2022). In addition, cefepime+VNRX-5133 (4–32 μg/ml+4 μg/ml) showed elevated MIC values against MBL-*P. aeruginosa* and its antibacterial effect against *P. aeruginosa*, with an emphasis on VIM producers, not NDM or SPM (Hamrick et al., 2020; Kloezen et al., 2021; Mushtaq et al., 2021; Hernandez-Garcia et al., 2022). No antibacterial activity was found for cefepime+ VNRX-5133 on MBL-producing *A. baumannii* (Mushtaq et al., 2021). In addition to being used in combination with cefepime, VNRX-5133 (4 μg/ml) can also be used in combination with meropenem (0.016–1 μg/ml) to play a considerable antibacterial role against the most MBL-positive Enterobacteriaceae (Wang et al., 2020).

Although there are no *in vivo* studies of cefepime+VNRX-5133 against MBLs in animal models, VNRX-5133 in combination with cefepime is currently in phase 3 clinical trials for the treatment of complex urinary tract infections (Dowell et al., 2021). We look forward to the formal clinical application of cefepime+VNRX-5133 in the near future.

### Diazabicyclooctanes alone and in combination with antibiotics

In the mid-1990s, chemists first studied diazabicyclooctanes (DBOs) as β-lactamase analogs and demonstrated that they were a rich source of β-lactamase inhibitors (Coleman, 2011). First, DBOs inhibit most or all class A and C β-lactamases, as well as some class D β-lactamases (Mushtaq et al., 2019). AVI is the sole analog of DBOs thus far licensed for clinical use and is combined with CAZ to treat complex intraperitoneal infections and complex urinary tract infections associated with gram-negative bacteria (Shirley, 2018). Since then, DBOs have been promising non-β-lactam inhibitors of β-lactamases (Shlaes, 2013).

Surprisingly, DBOs have also been reported to have anti-MBL activity in recent years (Livermore et al., 2015, 2017). Some novel DBOs not only have synergistic activity with certain antibiotics but also have direct antibacterial activity against MBL producers (Livermore et al., 2017). Several developmental DBOs, notably zidebactam, nacubactam, and WCK5153, have significant affinity for PBP2 of many gram-negative species (Livermore et al., 2015, 2017; Moya et al., 2017). This allows for the antibiotics to exert both a direct antibacterial effect and an “enhancer” mechanism, potentiating partner β-lactams that bind to other PBPs (Livermore et al., 2015, 2017; Moya et al., 2017). This dual function of direct and enhancer-based activity means that combinations of MBL-labile β-lactams with DBOs can retain activity against MBL producers (Mushtaq et al., 2019). These DBOs are reviewed in the following pages.

#### Zidebactam alone and in combination with antibiotics

Zidebactam, as a new DBO, exerts a dual function involving selectively high affinity for gram-negative bacterial PBP2 binding and β-lactamase inhibition (Bhagwat et al., 2019). By binding to PBP2 binding, zidebactam exhibits antibacterial activity against various Enterobacteriaceae and *P. aeruginosa* isolates (Sader et al., 2017). In a study conducted by Livermore *et al*., zidebactam alone at ≤1 μg/ml and at 8–32 μg/ml exerted *in vitro* antibacterial activity against most of the tested MBL-producing Enterobacteriaceae and MBL-producing *P. aeruginosa* (Livermore et al., 2017). For MBL-carrying Enterobacteriaceae and *P. aeruginosa* isolates, zidebactam not only has an antibacterial effect but can also be used as a synergistic agent with antibiotics. Then, more attention was given to zidebactam application as an antibiotic sensitizer, and several studies were carried out to evaluate the *in vitro* antibacterial activity of cefepime+zidebactam, often described as “WCK5222.” Cefepime+zidebactam (≤0.06–8 μg/ml+ ≤ 0.06–8 μg/ml) was proven to be effective against most of the tested MBL-producing Enterobacteriaceae isolates *in vitro* (Livermore et al., 2017; Moya et al., 2019; Avery et al., 2020; Karlowsky et al., 2020; Yang et al., 2020; Bhagwat et al., 2021; Vazquez-Ucha et al., 2022). Similar results were shown for MBL-producing *P. aeruginosa* isolates. Cefepime+zidebactam (≤0.25–16 μg/ml+4–16 μg/ml) can inhibit most of the tested MBL-producing *Pseudomonas* (mainly *P. aeruginosa*) isolates (Livermore et al., 2017; Moya et al., 2017; Monogue et al., 2019; Karlowsky et al., 2020; Kidd et al., 2020; Bhagwat et al., 2021). What we want to emphasize is that no obvious inhibitory effect of zidebactam or cefepime+zidebactam was found on MBL-producing *A. baumannii* isolates (Livermore et al., 2017; Yang et al., 2020). In addition to cefepime, zidebactam (4 μg/ml) can also combine with aztreonam (0.25–2 μg/ml) against MBL-producing *K. pneumoniae* and MBL-producing *P. aeruginosa* isolates, which once again proves the potential of zidebactam as an antibiotic sensitizer against MBLs (Moya et al., 2017, 2019).

*In vivo* studies using animal models also demonstrated the efficacy of cefepime+zidebactam or aztreonam+zidebactam against MBLs. In a neutropenic thigh mouse infection model caused by *P. aeruginosa* producing VIM, VIM+SBL, or NDM+SBL, 22-h log_10_ CFU/thigh for cefepime (2 g q8 h, 1-h infusion) plus zidebactam (1 g q8 h, 1-h infusion) was significantly lower than that for no drug therapy, cefepime (2 g q8 h, 1-h infusion) monotherapy, and zidebactam (1 g q8 h, 1-h infusion) monotherapy (Monogue et al., 2019). In neutropenic lung pneumonia mice infected with *P. aeruginosa* producing VIM, VIM+SBLs, or NDM+SBLs, 24-h log_10_ CFU/lung for cefepime (2 g q8 h, 1-h infusion) plus zidebactam (1 g q8 h, 1-h infusion) was significantly lower than that for no drug therapy, cefepime (2 g q8 h, 1-h infusion, s.c.) monotherapy, and zidebactam (1 g q8 h, 1-h infusion, s.c.) monotherapy (Kidd et al., 2020). In a neutropenic mouse thigh infection model caused by NDM+SBL-positive *K. pneumoniae*, 24-h log_10_ CFU/thigh for cefepime (100 mg/kg q2 h) plus zidebactam (75 mg/kg q2 h) was significantly lower than that for no drug therapy, meropenem-cilastatin (37.5 mg/kg q4 h) monotherapy, cefepime (100 mg/kg q2 h) monotherapy, and zidebactam (75 mg/kg q2 h) monotherapy; 24-h log_10_ CFU/thigh for aztreonam (75 mg/kg q2 h) plus zidebactam (75 mg/kg, q2 h) was significantly lower than that for no drug therapy, meropenem-cilastatin (37.5 mg/kg q4 h) monotherapy, aztreonam (75 mg/kg q2 h) monotherapy, and zidebactam (75 mg/kg q2 h) monotherapy (Moya et al., 2019).

These results support the clinical development of cefepime in combination with antibiotics for the treatment of bacterial infections caused by MBL-producing Enterobacteriaceae and *P. aeruginosa*. Additionally, the cefepime/zidebactam combination (intravenous dose regimen: q8 h, 1-h infusion) is slated to enter phase 3 studies for infections caused by MDR gram-negative bacteria (Bhatia and Wockhardt, 2022).

#### OP0595 (Nacubactam) alone and in combination with antibiotics

Nacubactam, another new DBO, is also reported to show *in vitro* antibacterial activity against Enterobacteriaceae with MBLs either alone or in combination with PBP3-targeted β-lactams. Livermore et al. found that OP0595 at 4 μg/ml not only inhibited most MBL-producing Enterobacteriaceae but also achieved ≥8-fold potentiation of piperacillin, cefepime, and biapenem for MBL-producing *K. pneumoniae* isolates (Livermore et al., 2015). However, OP0595 at 4 mg/L achieved no significant potentiation of aztreonam, biapenem, piperacillin, or cefepime against the MLB-producing *P. aeruginosa* and *A. baumannii* (Livermore et al., 2015). In another study, OP0595 at 4 μg/ml potentiated meropenem (1 μg/ml), cefepime (2 μg/ml), and aztreonam (1 μg/ml) in most of the tested NDM-producing *K. pneumoniae* isolates, respectively (Mushtaq et al., 2019). No *in vivo* study of OP0595 against MBL producers in animal models was found.

#### WCK5153 in combination with antibiotics

No positive inhibitory effect on MBLs was reported for WCK5153 alone, a novel DBO. Nevertheless, WCK5153 has been proven to be effective against MBL-producing *K. pneumoniae* and *P. aeruginosa* isolates *in vitro* when used in combination with several antibiotics. Moya et al. found that WCK5153 (4 μg/ml) can enhance the effectiveness of several antibiotics against MBL-producing *P. aeruginosa in vitro*, including cefepime, aztreonam, piperacillin, imipenem, meropenem, and doripenem (Moya et al., 2017). Moya et al. confirmed the *in vitro* efficacy of cefepime (≤0.25–0.5 μg/ml) or aztreonam (≤0.25–4 μg/ml)+WCK5153 (4 μg/ml) on MBL-producing *K. pneumoniae* using a microdilution broth method (Moya et al., 2019). These authors also confirmed the *in vivo* efficacy of these two combinations using a neutropenic mouse thigh infection model caused by NDM+SBL-positive *K. pneumonia*e. Briefly, the 24-h log_10_ CFU/thigh for cefepime (100 mg/kg q2 h) plus WCK5153 (75 mg/kg q2 h) was significantly lower than that for no drug therapy, meropenem-cilastatin (37.5 mg/kg q4 h) monotherapy, cefepime (100 mg/kg q2 h) monotherapy, and WCK5153 (75 mg/kg q2 h) monotherapy; the 24-h log_10_ CFU/thigh for aztreonam (75 mg/kg q2 h) plus WCK5153 (75 mg/kg q2 h) was significantly lower than that for no drug therapy, meropenem-cilastatin (37.5 mg/kg q4 h) monotherapy, aztreonam (75 mg/kg q2 h) monotherapy, and WCK5153 (75 mg/kg q2 h) monotherapy (Moya et al., 2019).

### Meropenem +ANT2681

ANT2681 is a specific, competitive inhibitor of MBLs with particularly potent activity against NDM, progressing to clinical development in combination with meropenem (Zalacain et al., 2021). Zalacain et al. verified the *in vitro* potent activity of meropenem+ANT2681 against NDM producers in a large population of Enterobacteriaceae strains (Zalacain et al., 2021). Meropenem+ANT2681 (≤0.06–1 μg/ml+8 μg/ml) can inhibit 80.7% of Enterobacteriaceae strains producing NDM, which is much higher than that producing VIM (29.1%) or IMP (10.2%). In addition, the antibacterial activity of meropenem+ANT2681 (≤0.06–1 μg/ml+8 μg/ml) against Enterobacteriaceae strains producing NDM+SBLs (10.6%) was much lower than that only producing NDM (80.7%), which likely indicated that meropenem+ANT2681 showed specific antibacterial activity against NDM-only Enterobacteriaceae. However, the specific potent antibacterial activity for NDM-only producers was not found in another study involving eleven Enterobacteriaceae strains, which may be related to the deviation of test results caused by too few strains (Davies et al., 2020). No study on meropenem+ANT2681 against MBL-producing *P. aeruginosa* or *A. baumannii* was found.

Regarding the *in vivo* efficacy, meropenem+ANT2681 was found to be effective against the NDM producer in a murine thigh infection model, which provides the underpinning evidence for meropenem+ANT2681 in clinical development. Specifically, in the neutropenic mouse thigh infection model caused by NDM-1-positive *E. coli*, the reduction of 24-h log_10_ CFU/thigh by meropenem (50 mg/kg q4 h) plus ANT2681 (89 mg/kg q4 h) was more than 1.5 log_10_ CFU/thigh in comparison to that by no drug therapy (Das et al., 2020).

### Meropenem+QPX7728

QPX7728 is a recently discovered ultra-broad-spectrum β-lactamase inhibitor from a class of cyclic boronates, with potent activity against MBLs (Hecker et al., 2020). It has been proved to be effective against MBL-producing gram-negative bacteria *in vitro* when in combination with meropenem. QPX7728 (4–8 μg/ml) restores sensitivity to meropenem for almost all tested MBL-positive Enterobacteriaceae strains (Nelson et al., 2020a; Lomovskaya et al., 2022). It was also found that QPX7728 (8 μg/ml) restored sensitivity to meropenem for some NDM-positive *Acinetobacter* strains (Nelson et al., 2020b). Compared with *Acinetobacter*, this combination may have a weaker effect on MBL-positive *P. aeruginosa*. Meropenem+QPX7728 (≤8+8 μg/ml) was found to be effective in only one-third of the MBL-positive *P. aeruginosa* strains (Lomovskaya et al., 2021). No *in vivo* study of this combination against MBL producers in animal models was found.

### Eravacycline

Eravacycline is a novel and fully synthetic fluorocycline antibacterial agent. Its structure is similar to that of tigecycline, with two modifications to the D-ring of its tetracycline core: a fluorine atom replaces the dimethylamine moiety at C-7, and a pyrrolidinoacetamido group replaces the 2-tertiary-butyl glycylamido at C-9 (Zhanel et al., 2016). Recently, eravacycline has been approved for use in complicated intra-abdominal infections, including infections caused by MBL producers (Solomkin et al., 2019). Eravacycline at 1–4 μg/ml exerts potent antibacterial activity on all or most of the tested Enterobacteriaceae strains producing MBLs (Zhang et al., 2016; Johnston et al., 2020; Maraki et al., 2022). However, no relevant studies have been conducted to test its antibacterial activity in MBL-producing *P. aeruginosa* or *A. baumannii* thus far.

### Plazomicin

Plazomicin is an aminoglycoside developed from a sisomicin scaffold *via* chemical modification to evade the most common aminoglycoside resistance mechanisms in Enterobacteriaceae, such as β-lactamases, including MBLs, as well as fluoroquinolone and colistin resistance mechanisms (Castanheira et al., 2018). For this reason, plazomicin maintains its activity against many MDR Enterobacteriaceae and was approved in 2018 by the FDA for the treatment of complicated urinary tract infections related to *E. coli, K. pneumoniae, Enterobacter cloacae*, and *Proteus mirabilis* (Serio et al., 2019). Several studies *in vitro* have confirmed its antibacterial activity on MBL-producing Enterobacteriaceae at a concentration of 0.75–2 μg/ml (Serio et al., 2019; Johnston et al., 2021; Maraki et al., 2022). No study on its antibacterial activity against MBL-producing *P. aeruginosa* or *A. baumannii* was found.

### KBP-7072

KBP-7072 is an effective, broad-spectrum, third-generation tetracycline (aminomethylcycline) antimicrobial agent under development (oral and intravenous formulations) for the treatment of community-acquired pneumonia, overcoming many common mechanisms of tetracycline resistance (Lepak et al., 2019). KBP-7072 shows excellent antibacterial activity against MBL-positive *A. baumannii* isolates with MIC values ranging from 0.12 to 1 μg/ml *in vitro*, which is significantly better than that of the comparator agents (MIC: 0.5–32 μg/ml), including tigecycline, ceftazidime, gentamicin, levofloxacin, and meropenem (Huband et al., 2020b). No other study on its antibacterial activity against MBL-producing Enterobacteriaceae or *P. aeruginosa* was found.

### LYS228

LYS228 is a novel monobactam with potent antibacterial activity against carbapenem-resistant Enterobacteriaceae and is stable to MBLs. LYS228 at ≤0.06–4 μg/ml exerts potent *in vitro* antibacterial activity on most of the tested Enterobacteriaceae strains producing MBLs, which is obviously more active than aztreonam, ceftazidime, ceftazidime/avibactam, cefepime, and meropenem (Blais et al., 2018). Weiss et al. confirmed its *in vivo* efficacy using a neutropenic mice thigh infection model caused by NDM-1 positive *K. pneumonia*e. In brief, 24-h log_10_ CFU/thigh for LYS228 (90 mg/kg q4 h) was significantly lower than that for no drug therapy (Weiss et al., 2019). No study on its antibacterial activity against MBL-producing *P. aeruginosa* or *A. baumannii* was found.

## Non-antibacterial drugs

Studies have shown that some non-antibacterial drugs also have antibacterial activity. According to a study published in *JAMA Cardiol*, the conventional antiplatelet dose of ticagrelor had good antibacterial activity against gram-positive bacteria (Lancellotti et al., 2019). Its bactericidal effect against methicillin-resistant *Staphylococcus aureus* and methicillin-resistant *Staphylococcus epidermidis* was better than that of vancomycin, and its bactericidal effect against methicillin-resistant *S. aureus* and vancomycin-resistant *Enterococcus* was similar to that of daptomycin (Lancellotti et al., 2019). Beyond that, non-antibacterial drugs with antibacterial activity to MBL producers have also been reported in recent years.

Captopril, a classical antihypertensive drug, chelates prosthetic groups and destroys the catalytic activity of MBL (Heinz et al., 2003). Captopril is reported to effectively inhibit NDM-1 with an IC50 value of 7.9 μM (Li et al., 2014). An *in vitro* study showed that captopril (10–320 μg/ml) potentiated meropenem activity and restored its efficacy against MBL-producing *K. pneumoniae* (Zhao et al., 2021). In *Galleria mellonella* infected by IMP-4-positive *K. pneumoniae*, the survival rate for captopril (25 mg/kg) plus meropenem (10 mg/kg) was significantly higher than that for no drug therapy, captopril (25 mg/kg) monotherapy, and meropenem (10 mg/kg) monotherapy; 24-h CFU/larva for captopril plus meropenem was significantly lower than that for no drug therapy and meropenem monotherapy (Zhao et al., 2021). Especially, captopril analogs based on a series of structure-guided designs that can inhibit the activity of MBLs are being discovered (Li et al., 2014; Yusof et al., 2016; Ma et al., 2021).

Ebselen is a drug used to treat cerebral ischemia and stroke in human clinical trials. Chiou et al. found that ebselen is capable of covalently binding to the Cys221 residue in the active site of the NDM-1 enzyme, which ultimately results in the loss of one zinc ion (Chiou et al., 2015). *In vitro*, ebselen alone has low intrinsic antibacterial activity but can reduce the MICs of ampicillin and meropenem by 16- and 128-fold, respectively, against NDM-1-positive *E. coli* (detailed data not presented) (Jin et al., 2020). Compared with ebselen, a 2-substituted 1,2-benzisothiazol-3(2H) derivative of ebselen exhibited much stronger activity against NDM-1 isolates. This derivative (4–32 μg/ml) in combination with meropenem (4–32 μg/ml) demonstrated potent synergistic activity against NDM-1-positive Enterobacteriaceae (Jin et al., 2020).

## Antimicrobial peptides

Antimicrobial peptides (AMPs), also known as host defense peptides, can be found virtually in every life form, from prokaryotes to humans. To date, there have been many databases of natural AMPs, consisting of more than 2,000 peptides and exhibiting remarkable functional diversity (Wang, 2015). They not only possess direct and broad-spectrum antimicrobial activity but also are capable of modulating innate immunity in higher organisms to protect hosts from infectious diseases (Hancock and Sahl, 2006; Pasupuleti et al., 2012). Currently, AMPs have been reported to be effective against MBL-producing Enterobacteriaceae.

Thanatin is a 21-residue antimicrobial peptide with a disulfide bond between Cys11 and Cys18 that exerts potent activity against NDM-1-producing bacteria by a dual mechanism of action: disrupting the outer membrane of NDM-1 producers and inhibiting the enzymatic activity of NDM-1 by displacing zinc ions from the active site (Ma et al., 2019). It was found that thanatin (1–8 μg/ml) could exhibit a potent inhibitory effect on NDM-1-producing Enterobacteriaceae strains *in vitro* (Soren et al., 2015; Ma et al., 2019). The *in vivo* therapeutic effects of thanatin were confirmed in a sepsis model. In the sepsis mouse model caused by NDM-1-positive *E. coli*, the survival rates for thanatin (1, 3, or 6 mg/kg) increased by 30–100% compared with that for no drug therapy; 23-h log_10_ CFU/tissues (blood, lung, liver, and spleen) for thanatin was significantly lower than that for no drug therapy; obvious rescued pathological damages were found for thanatin, in comparison to no drug therapy (Ma et al., 2019). In addition, thanatin (concentration not described) exerted synergistic or additive effects with imipenem and meropenem against the NDM-1-producing *K. pneumoniae* strain *in vitro* (Ma et al., 2019). Similarly, the *in vivo* therapeutic effects of thanatin+meropenem were confirmed in a sepsis model. In the sepsis mouse model caused by NDM-1-positive *E. coli*, the survival rate for thanatin (0.1 mg/kg) plus meropenem (10 mg/kg) increased by 49–100% compared with that for no drug therapy, thanatin (0.1 mg/kg) monotherapy, and meropenem (10 mg/kg) monotherapy; 23-h log_10_ CFU/tissues (spleen and liver) in target regimen was significantly lower than that for no drug therapy, thanatin monotherapy, and meropenem monotherapy (Ma et al., 2019).

Novicidin, a novel 18-residue cat-ionic antimicrobial peptide, exerts bactericidal effects by disrupting the bacterial membrane (Dorosz et al., 2010; Nielsen and Otzen, 2010). The combination of novicidin with rifampin was shown to have synergistic activity against all tested NDM-1 Enterobacteriaceae strains *in vitro* (Soren et al., 2015).

## Natural products

Natural products from microorganisms and plants have been a rich source of starting materials in the search for new drugs. Natural products may represent potential lead compounds for anti-MBL drug development. Natural products serve as natural antibiotics to make multidimensional strategies against MBLs more intact, which is of clinical importance.

Aspergillomarasmine A (AMA) is a fungal natural product considered a rapid and potent MBL inhibitor that electively sequesters Zn^2+^ (King et al., 2014; Sychantha et al., 2021). AMA itself is not antimicrobial but works in combination with β-lactam antibiotics or carbapenems against MBL producers, with an emphasis on VIM or NDM. King et al. found that AMA (8 μg/ml) fully restored the activity of meropenem against >88% of Enterobacteriaceae, *Acinetobacter* spp. and *Pseudomonas* spp. strains possessing either VIM or NDM (King et al., 2014). Rotondo et al. found that AMA (≤0.5–64 g/ml) could be used not only with carbapenems but also with β-lactam antibiotics against MBL producers Enterobacteriaceae, similar to an emphasis on VIM or NDM (Rotondo et al., 2020). Iminodiacetic acid, as the metal-binding pharmacophore core of AMA, has been used for fragment-based drug discovery of NDM-1 inhibitors (Chen et al., 2020). Emerione A, identified from fungal products by *in silico* screening, is the second fungal metabolite reported to exhibit NMD-1 inhibitory activity after AMA. Emerione A (8–128 μg/ml) potentiates the activity of four β-lactam antibiotics against two kinds of NDM-1-producing Enterobacteriaceae (He et al., 2022).

Rosmarinic acid is widely identified in the plant kingdom, such as the plant families Lamiaceae, Boraginaceae, and Marantaceae. Kinetic assays revealed that rosmarinic acid is a fully reversible, substrate-competitive VIM-2 inhibitor (Yu et al., 2018). Salvianolic acid A, a derivative of RA, manifests potent inhibition of VIM-2, more interestingly, which also shows inhibitory activity against NDM-1 (Yu et al., 2018). Both rosmarinic acid and salvianolic acid A resulted in a 2- to 4-fold MIC reduction of meropenem against VIM-2-producing *E. coli* (Yu et al., 2018).

Some other natural products, which have been reported to have anti-MBL activity at the enzymatic level but have not been studied at the cellular level, will not be described here, such as alcohol extracts of *Schisandra chinensis* and baicalin (Shi et al., 2019; Duan et al., 2021; Salari-Jazi et al., 2021). In addition, natural products with very limited anti-MBL activity are no longer described here, such as the aquo-ethanolic extract of *Camellia sinensis* (Thakur et al., 2016) and the methanolic extract of *Punica granatum* (Dey et al., 2012).

## Zinc chelators

The zinc-dependent activity of MBL allows its inhibition by zinc-chelating agents. Several compounds have potential usage to treat MBL producers in combination with existing antibiotics.

Two zinc chelators, N, N, N′, N′-tetrakis (2-pyridylmethyl) ethylenediamine (TPEN) and di-(2-picolyl) amine (DPA), were able to inhibit MBLs and render MBL producers susceptible to meropenem (Azumah et al., 2016). TPEN at 46–32 μg/ml and DPA at 8–32 μg/ml regain susceptibility to meropenem for most tested Enterobacteriaceae strains harboring MBL (Azumah et al., 2016). 1,4,7-Triazacyclononane-1,4,7-triacetic acid (NOTA) is a superior chelator of copper and gallium (Boros et al., 2010; Zhang et al., 2011). It is found to chelate zinc, which may be due to zinc having a size between that of copper and gallium (Somboro et al., 2015). NOTA (4 μg/ml) was able to restore the activities of carbapenems against most tested MBL-producing Enterobacteriaceae strains. TACN, a cyclic organic compound within NOTA derived from cyclononane by the replacement of three equidistant CH_2_ groups with NH groups, significantly reduced the MIC of meropenem against MBL-producing Enterobacteriaceae strains (Somboro et al., 2019). In brief, the combination of meropenem (0.06–8 μg/ml) + TACN (4–8 μg/ml) was synergistic for all tested MBL-producing Enterobacteriaceae strains.

Ca-EDTA, a metallo-chelator, was created as an injectable form of chelator with low toxicity, which has been approved for the treatment of lead intoxication (Lin-Tan et al., 2007). Aoki et al. and Yoshizumi et al. successively reported the efficacy of Ca-EDTA as an inhibitor of MBL (Aoki et al., 2010; Yoshizumi et al., 2013). The addition of Ca-EDTA (32 μg/ml) drastically reduced the MICs of carbapenems (imipenem, meropenem, and amikacin) in a small proportion of MBL-positive Enterobacteriaceae and *P. aeruginosa in vitro* (Aoki et al., 2010; Yoshizumi et al., 2013). The *in vivo* efficacy of combining Ca-EDTA (200–300 mg/kg q.d.) with IPM (50 mg/kg/day) against MBL-positive *P. aeruginosa* was confirmed in a pneumonia mouse model. In the mouse model of pneumonia caused by IMP-1-positive *P. aeruginosa*, the survival rate for mice treated with Ca-EDTA (200 mg/kg q.d. or 300 mg/kg q.d.) plus imipenem (50 mg/kg q.d., s.c.) increased by ~70–100% compared with that for mice given no drug therapy, Ca-EDTA (200 mg/kg q.d or 300 mg/kg q.d.) monotherapy, and imipenem (50 mg/kg q.d.) monotherapy; the reduction in bacterial burden by Ca-EDTA plus imipenem was more than 1 log_10_ CFU/lung in comparison to that by no drug therapy and imipenem monotherapy (Aoki et al., 2010). Moreover, these *in vivo* data suggest the therapeutic potential of Ca-EDTA not only by blocking MBLs but also by neutralizing tissue-damaging metalloproteases in infections caused by *P. aeruginosa* (Aoki et al., 2010). In the acute lethal mouse septicemia model caused by NDM-1-positive *E. coli*, CFU/liver (not CFU/blood) was significantly reduced by Ca-EDTA (200 mg/kg) plus imipenem/cilastatin sodium (25 mg/kg) compared with that by no drug therapy, Ca-EDTA (200 mg/kg) monotherapy, and imipenem/cilastatin sodium (25 mg/kg) monotherapy (Yoshizumi et al., 2013). Furthermore, in a more severe model of infection (neutropenic sepsis mouse model) caused by NDM-1-positive *E. coli*, both CFU/liver and CFU/blood were significantly reduced by Ca-EDTA (100 mg/kg) plus imipenem/cilastatin sodium (10 mg/kg) compared with that by no drug therapy, Ca-EDTA (100 mg/kg) monotherapy, and imipenem/cilastatin sodium (10 mg/kg) monotherapy (Yoshizumi et al., 2013).

## Other compounds with MBL inhibitory activity

As mentioned above, antibiotic resistance as a global threat has aroused widespread concern, indicating that we have entered the “postantibiotic era.” To combat resistance crises, it is crucial to adopt multidimensional strategies.

Farley et al. synthesized nine N-sulfamoylpyrrole-2-carboxylates (NSPCs), which exert particularly potent activity against NDM-1 and IMP-1 (Farley et al., 2021). Crystallography reveals that the N-sulfamoyl NH_2_ group of NSPCs displaces the dopamine bridging hydroxide/water of the B1 MBLs. At a fixed concentration of 2 μg/ml, any one of the nine NSPCs reduced the meropenem MIC from 256 to ≤0.25–5 μg/ml in NDM-1 Enterobacteriaceae.

ML302 is a rhodamine-based MBL inhibitor. Mechanistic studies show that ML302 undergoes hydrolysis to yield a thioenolate fragment (ML302F), which triggers the formation of a ternary complex among the MBL, intact ML302, and ML302F by zinc chelation to potentiate antibiotic efficacy through collaborative strategies. ML302 (10 μg/ml) conferred meropenem sensitivity on IMP producers of Enterobacteriaceae, not NDM or VIM producers (Brem et al., 2014).

KHP-3757 is the most recent LpxC inhibitor of the hydroxamate class currently being evaluated in preclinical development studies (Huband et al., 2020a). Huband et al. demonstrated that KHP-3757 (0.06–0.5 μg/ml) had potent *in vitro* activity against all tested MBL-producing *P. aeruginosa* isolates, outperforming nine comparator agents, including amikacin, aztreonam, cefepime, ceftazidime, ciprofloxacin, colistin, imipenem, meropenem, and piperacillin-tazobactam (Huband et al., 2020a).

Khan et al. have reported the discovery of four novel non-β-lactam inhibitors (M1, M17, M21, and M61) against NDM-1 by a multi-step virtual screening approach (Khan et al., 2017). For *E. coli* producing NDM-1, the lower MICs were obtained when each of these inhibitors was combined with ceftazidime, cefoxitin, meropenem, and imipenem. The MICs were lowered from 8 to 4 μg/ml (ceftazidime), 16 to 8 μg/ml (cefoxitin), and 2 to 1 μg/ml for meropenem and imipenem.

## Discussion and future perspectives

Bacterial infections are one of the leading causes of human death worldwide. International antibacterial treatments mainly use antibiotics to inhibit the reproduction and growth of bacteria and can achieve efficient sterilization effects. β-Lactams have long played a vital role in the treatment of pathogenic bacterial infections. However, widespread and improper use of antibiotics for decades in turn has led to resistance in bacteria. The major resistance mechanism is the emergence of β-lactamases, especially the most relevant MBLs, which can hydrolyze the β-lactam ring. Thus, MBLs are capable of inactivating almost all β-lactams, with the exception of aztreonam. Nevertheless, the use of aztreonam was also limited by the fact that MBL-producing strains carried SBLs, which requires broad-spectrum inhibitors (Cervino et al., 2021). Therefore, the development of new drugs against MBL or MBL +SBL producers has become a global biomedical research hotspot.

This article reviews the current research progress on novel drugs, compounds, and drug combinations against MBL producers. As shown in Table 1, many novel drugs, compounds, and drug combinations with anti-MBL activity have not been studied in detail. For example, antimicrobial activity studies of some novel drugs have not included *P. aeruginosa* and *A. baumannii* that produce MBLs, and most compounds in this review have not been studied *in vivo*. It must be noted that the traditional development of antibiotics inevitably leads to the emergence of new resistant strains, making new drugs quickly ineffective. Developing a single inhibitor of MBLs is also technically too difficult, and overcoming *in vivo* toxicity due to cross-reactions with human metalloenzymes has been a concern. Therefore, restoring the therapeutic potential of existing antibiotics through drug combinations may be a very attractive strategy to solve clinically challenging MBLs. In this study, several drugs or compounds in this review that have not been studied for their synergistic effects on existing antibiotics should be complemented with further experimental data.

In addition, some of these novel drugs, compounds, and drug combinations have many desirable antimicrobial properties for the treatment of MBL-associated infections, but more *in vivo* efficacy and safety data are needed to fully determine their role in the treatment of infectious diseases. Therefore, further experimental data should be improved based on Table 2 to obtain stronger evidence of the efficacy and safety of MBL producers for these novel drugs, compounds, and drug combinations. Indeed, we are still encountering many setbacks on these roads, such as complicated preparation processes, uncertain PK and PD properties, and differential efficacy *in vitro* and *in vivo*.

Nevertheless, the evolution of MBLs never ends, which urges us to conduct continuing research. The field of antimicrobial resistance is a dynamic and rapidly evolving field, and the treatment of antimicrobial resistance will continue to challenge clinicians. The application of bioinformatics software tools and compound databases also holds great promise for the production of effective inhibitors against MBLs, which is not described in detail in this review.

Limitations to this study include that most of the drugs involved in this study only performed MIC measurements. Except for a few drugs tested for affinity against the MBLs, scarcely any of the other drugs have been studied for the mechanism of drug resistance against MBL producers. Therefore, we did not show the mechanisms, but it is hoped that this study will inspire more researchers to study the mechanism of these drugs.

In conclusion, these novel drugs, compounds, and their combinations with anti-MBL activity provide more therapeutic alternatives against complicated infections caused by MBLs, although further rigorous optimization is required for their clinical development. It is hoped that this review can provide ideas and enlightenment for others to study the methods against metalloenzymes and improve the existing research data to obtain convincing evidence.

## Author contributions

XL, HW, and YZ designed the concept and wrote the manuscript. BZ and XD assisted in making tables. JJ and WW finalized and corrected the manuscript. JZ assisted in the revision of the manuscript. All authors read and approved the final manuscript.

## Funding

This work was supported by the Shandong Provincial Natural Science Foundation [Grant no. ZR2020QH365], the Hospital Level Project of Shandong Province Maternal and Child Health Hospital [Grant no. 2021SFF062], and Shandong Province Medical and Health Science and Technology Development Plan [Grant no. 202006021519]. The funders will have no role in the study design, data collection and analysis, decision to publish, or preparation of the manuscript.

## Conflict of interest

The authors declare that the research was conducted in the absence of any commercial or financial relationships that could be construed as a potential conflict of interest.

## Publisher's note

All claims expressed in this article are solely those of the authors and do not necessarily represent those of their affiliated organizations, or those of the publisher, the editors and the reviewers. Any product that may be evaluated in this article, or claim that may be made by its manufacturer, is not guaranteed or endorsed by the publisher.

## Abbreviations

*P. aeruginosa*: *Pseudomonas aeruginosa*
*A. baumannii*: *Acinetobacter baumannii*
SBL: serine β-lactamases
MBL: metallo-β-lactamases
NDM: New Delhi metallo-β-lactamase
VIM: Verona imipenemase
IMP: imipenemase
CAZ: ceftazidime
AVI: avibactam
*E. coli*: *Escherichia coli*
*K. pneumoniae*: *Klebsiella pneumoniae*
FDA: Food and Drug Administration
DBO: diazabicyclooctane
AMP: antimicrobial peptides
AMA: Aspergillomarasmine A.
